# Disaccharide-tag for highly sensitive identification of O-GlcNAc-modified proteins in mammalian cells

**DOI:** 10.1371/journal.pone.0267804

**Published:** 2022-05-23

**Authors:** Hirohito Abo, Masahiko Kume, Federico Pecori, Taichi Miura, Naoki Matsumoto, Shoko Nishihara, Kazuo Yamamoto

**Affiliations:** 1 Faculty of Integrated Biosciences, Graduate School of Frontier Sciences, The University of Tokyo, Kashiwa, Chiba, Japan; 2 Department of Bioinformatics, Graduate School of Engineering, Soka University, Hachioji, Tokyo, Japan; 3 Glycan & Life System Integration Center (GaLSIC), Soka University, Hachioji, Tokyo, Japan; Fisheries and Oceans Canada, CANADA

## Abstract

O-GlcNAcylation is the only sugar modification for proteins present in the cytoplasm and nucleus and is thought to be involved in the regulation of protein function and localization. Currently, several methods are known for detecting O-GlcNAcylated proteins using monoclonal antibodies or wheat germ agglutinin, but these methods have some limitations in their sensitivity and quantitative comparison. We developed a new disaccharide-tag method to overcome these problems. This is a method in which a soluble GalNAc transferase is expressed intracellularly, extended to a disaccharide of GalNAc-GlcNAc, and detected using a *Wisteria japonica* agglutinin specific to this disaccharide. We verified the method using human c-Rel protein and also highly sensitively compared the difference in O-GlcNAc modification of intracellular proteins associated with differentiation from embryonic stem cell (ESC) to epiblast-like cells (EpiLC). As one example of such a modification, a novel O-GlcNAc modification was found in the transcription factor Sox2 at residue Ser263, and the modification site could be identified by nano liquid chromatography-mass spectrometry.

## Introduction

Glycosylation is the most sophisticated post-translational modification of proteins. N-glycosylation and O-glycosylation of proteins frequently occur on secreted and membrane-bound proteins, and these oligosaccharides play important roles in several biological phenomena, including intracellular and extracellular trafficking of glycoproteins, cell-cell or cell-matrix mediating, and modulating cell adhesion [1]. In contrast, modification of serine or threonine by O-linked β-N-acetylglucosamine (O-GlcNAcylation) occurs on nuclear or cytoplasmic proteins [2,3]. This modification is significantly different from N-glycosylation or O-glycosylation from several perspectives. First, O-GlcNAcylation occurs in the nucleus or cytoplasm of the cell, whereas general glycosylation occurs in the luminal regions of the endoplasmic reticulum and Golgi apparatus. Second, O-GlcNAcylation is a reversible reaction via the actions of O-GlcNAc transferase (OGT) and O-GlcNAcase (OGA), although N- and O-glycosylation are irreversible. Third, general N- and O-glycans on proteins are sequentially processed and elongated with the help of many types of glycosyltransferases. However, O-GlcNAc residues on nuclear or cytoplasmic proteins cannot be modified by other monosaccharides. Based on these biochemical properties of O-GlcNAcylation, it is clear that the biological function of O-GlcNAcylation is significantly different from that of N-glycosylation or O-glycosylation of proteins.

To detect O-GlcNAcylation of nuclear or cytoplasmic proteins, monoclonal antibodies against O-GlcNAc residues, named RL2 and CTD110.6, are widely used. However, these monoclonal antibodies recognize both O-GlcNAc residue and peptide portions; thus, the reactivities of these antibodies largely depend on the peptide sequences that are modified with O-GlcNAc residues. Wheat germ agglutinin (WGA) is also used to detect O-GlcNAcylated proteins because WGA binds to GlcNAc [4]. However, WGA preferentially binds to N-acetyl groups of both GlcNAc and N-acetylneuraminic acid residues attached to N- and O-linked oligosaccharides [5]; thus, such a probe is not suitable for comprehensive analysis of O-GlcNAcylated proteins. Binding modes of WGA to GlcNAc or to N-acetylneuraminic acid are different and N-acetylneuraminic acid, especially clustered sialic acids, binds strongly to WGA through ionic interaction between COOH groups of N-acetylneuraminic acids and basic amino acids on the surface of WGA. By succinylation of WGA, COOH groups are introduced into ε-NH_2_ groups of Lys, OH groups of Tyr, SH groups of Cys, and OH groups of Ser/Thr, and the pI value changes from 8.5 to 4.0. Such chemical modification leads to WGA binds only to GlcNAc [6]. Therefore, succinylated WGA is used to specifically detect O-GlcNAcylated proteins [7].

To overcome such weak points of these O-GlcNAc probes, we established a novel method to detect specifically and with high sensitivity, O-GlcNAcylated proteins by expressing soluble β-GalNAc transferase in the nucleus or cytoplasm of cells. In this method, O-GlcNAc residues are converted to disaccharide GalNAc-GlcNAc, and many O-GlcNAcylated proteins were detected by *Wisteria japonica* agglutinin (WJA), which specifically recognizes GalNAcβ1-3(4)GlcNAc sequences, as reported previously [8].

## Materials and methods

### Cell lines and culture

HEK293 and HEK293T cells were obtained from the Cell Resource Center for Biochemical Research (Tohoku University, Miyagi, Japan) and maintained in Dulbecco’s modified Eagle’s medium (Sigma-Aldrich, St. Lewis, MO) supplemented with 10% heat-inactivated fetal calf serum (FCS, Invitrogen, Carlsbad, CA), 100 μg/ml penicillin, 100 U/ml streptomycin, 2 mM glutamine, 25 mM HEPES, and 50 mM 2-mercaptoethanol under 5% CO_2_ at 37 ˚C. R1 mouse ES cell lines were maintained on mouse embryonic fibroblasts inactivated with 10 mg/mL mitomycin C (Sigma-Aldrich) in ESC medium consisting of DMEM supplemented with 15% fetal bovine serum (Nichirei Biosciences, Tokyo, Japan), 1% penicillin/streptomycin (Gibco), 0.1 mM 2-mercaptoethanol (Gibco), 0.1 mM nonessential amino acids (Gibco), and 1000 U/mL LIF (Chemicon International, Temecula, CA).

### Expression of soluble β-N-acetylgalactosaminyltransferase in HEK293T, Yama-paca-6, and ES cells

Construction of expression plasmids for soluble β4GalNAc transferase from humans or Drosophila was performed as described below. A cDNA encoding human β4GalNAc transferase was kindly gifted by Dr. Takashi Sato of the National Institute of Advanced Industrial Science and Technology (AIST, Tsukuba, Japan) and Drosophila β4GalNAc transferase-A, as described previously [9]. To express the soluble Drosophila β4GalNAc transferase-A (β4GalNAc-TA), cDNA encoding residues from 62 to 403 were amplified by PCR using the primers 5’-a*gaattc*aggaggcagtgaagcatccag-3’ and 5’-a*ggatcc*ctaacttttgcgctcggagt-3’, which contain restriction sites for EcoR I and BamH I, respectively (shown in italic font). Beta1,4-N-acetylgalactosaminyl transferase containing a nuclear localization signal (NLS), PPKKKRKV, from SV40 T antigen, at the N- or C-terminus were also generated by PCR using 5’-ttaggaattcaccaccaaaaaagaagagaaaggttggaggcagtgaagcatccag-3’ and 5’-tagggatccctaaacctttctcttcttttttggtggacttttgcgctcggagttc-3’ as forward and reverse primers, respectively. Each amplified DNA was inserted between the Sma I site of the pBluescript SK II (+) vector, and its nucleotide sequence was confirmed. pBluescript SK II(+)-soluble β4GalNAc-TA was digested with EcoR I and BamH I and then inserted between the EcoR I and BamH I sites of the pFLAG-CMV3 (Sigma-Aldrich) expression vector to fuse the recombinant protein to a FLAG-tag at the N-terminus. These constructed plasmids were transfected into HEK293T, Yama-paca-6, or ES cells using Lipofectamine 2000 reagent (Invitrogen, Carlsbad, CA) according to the manufacturer’s protocol. Differentiation from ESC to EpiLC was performed as previously described [10]. ESCs transfected with β4GalNAc-TA were cultured in ESC medium in the presence of 1 mM PD0325901 (Wako, Tokyo, Japan), 3 mM CHIR99021 (Wako), and LIF. Twenty four hours after puromycin selection, EpiLC medium, consisting of DMEM/F12 (Gibco) supplemented with 20% knockout serum replacement (Gibco), 2mM L-glutamine (Invitrogen), 1% penicillin/streptomycin (Gibco), 0.1 mM 2-mercaptoethanol (Gibco), 0.1 mM nonessential amino acids (Gibco), 30 ng/ml FGF2 (Wako) and 0.6 mM JAK inhibitor (JAKi) (Santa Cruz Biotechnology, Dallas, TX), was added. EpiLC medium was changed daily and EpiLCs were collected for O-GlcNAcylated protein analysis after 96 h. Experiments using human materials and genetic recombination experiments were conducted in accordance with a comprehensive, high quality care program, which has been approved by the Life Science Research Committee of the Graduate School of Frontier Sciences of The University of Tokyo guided by the Life Science Committee of The University of Tokyo.

### Assay of soluble FLAG-tagged β4GalNAc-TA activity *in vitro*

β4GalNAc-TA activity was measured by incubating recombinant FLAG-tagged β4GalNAc-TA and FLAG-tagged human c-Rel in 14 mM HEPES-NaOH, pH 7.4, containing 0.2% Triton X-100, 10 mM MnCl_2_, 0.2 mM UDP-GalNAc, at 25°C for 12 h. After incubation, 6% SDS, 30% glycerol, and 2% DTT were added to the reaction mixture, which was then electrophoresed on 10% polyacrylamide gels. After Western blotting on Immobilon P membrane, the membrane was stained with anti-FLAG-Ab and WJA lectin, as described above.

### Immunoprecipitation and pull-down assay

Cells were lysed with lysis buffer (50 mM Tris-HCl, pH 7.4, 150 mM NaCl, 1% Triton X-100, 5 mM EDTA, 1 mM Na_3_VO_4_, 10 mM NaF, and a protease inhibitor cocktail (Sigma-Aldrich). A sample (200–1000 μg) of proteins was diluted 10-fold with wash buffer (50 mM Tris-HCl, pH 7.4, 150 mM NaCl, 5 mM EDTA, 1 mM Na_3_VO_4_, 10 mM NaF, and a protease inhibitor cocktail). For immunoprecipitation, anti-Sox2 antibody (Ab) (Santa Cruz Biotechnology), anti-FLAG Ab (Sigma-Aldrich), anti-O-GlcNAc Ab RL2 clone (Santa Cruz Biotechnology), normal mouse IgG (Santa Cruz Biotechnology), or normal rabbit IgG (R&D Systems, Minneapolis, MN) were added to the diluted cell lysate. Protein G magnetic beads (New England Biolabs, Ipswich, MA) were then added. For the pull-down assay, the diluted cell lysate was incubated with succinylated wheat germ agglutinin-biotin (sWGA-biotin, EY Laboratories, San Mateo, CA), streptavidin magnetic beads (Bio-Rad, Hercules, CA) were then added. The precipitated fractions were then washed five times with wash buffer.

### Western blotting

Cells were lysed with lysis buffer (50 mM Tris-HCl, pH 7.4, 150 mM NaCl, 1% Triton X-100, 5 mM EDTA, 1 mM Na_3_VO_4_, 10 mM NaF, and protease inhibitors). Protein samples were separated on an SDS polyacrylamide gel and transferred to polyvinylidene fluoride membranes (Millipore, Burlington, MA). Membranes were blocked using 1% BSA and incubated with the following primary antibodies: anti-Sox2 antibody (R&D Systems), anti-FLAG antibody (Sigma-Aldrich), anti-O-GlcNAc antibody RL2 clone (Santa Cruz), and anti-β-actin antibody (Sigma-Aldrich). The membranes were then incubated with anti-rabbit IgG (light chain specific, Jackson Immunoresearch, West Grove, PA) or anti-mouse IgG (Cell Signaling, Danvers, MA) conjugated with horseradish peroxidase (HRP) as a secondary antibody. The membranes were then washed and developed with ECL Plus reagents (GE Healthcare, Chicago, IL).

### Two-dimensional gel electrophoresis and Western blotting by *Wisteria japonica* agglutinin

HEK293T cells transformed with pFLAG-CMV3-β4GalNAc-TA, pFLAG-CMV3-NLS-β4GalNAc-TA, or pFLAG-CMV3-β4GalNAc-TA-NLS were prepared using Lipofectamine 2000 (Thermo Fisher Scientific, Waltham, MA). For two-dimensional electrophoresis, transfected cells were lysed with 7 M urea, 2 M thiourea, 2% 3[(3-cholamidopropyl) dimethylammonio]-1-propanesulfonate (CHAPS), 2% sulfobetaine 10, 1% protease inhibitor cocktail (Sigma-Aldrich), and 65 mM dithiothreitol (DTT), and then subjected to two-dimensional gel electrophoresis. Two-dimensional gel electrophoresis was performed according to a previously described method [11]. Briefly, isoelectric focusing was carried out on Immobiline DryStrips (pH 4–7, 18 cm; Amersham Biosciences, Buckinghamshire, UK) using CoolPhoreStar IPG-IEF type-P (Anatech, Tokyo, Japan) for 18 h. The strips were equilibrated in 50 mM Tris-HCl (pH 6.8, containing 6M urea, 2% SDS, 30% glycerol, and 2% DTT, and then electrophoresed on 10% polyacrylamide gels according to the method described by Laemmli [12]. For the *Wisteria japonica* agglutinin (WJA) staining, two-dimensional gels were transferred to Immobilon P membrane (Millipore) and the membrane was stained with 0.1 mg/mL of biotin-labeled WJA, followed with peroxidase-conjugated streptavidin. Binding of WJA was visualized using ECL Western blotting detection reagent (GE Healthcare) according to the manufacturer’s instructions.

### Identification of O-GlcNAcylated proteins using mass spectrometry (MS)

Several areas on the gel were excised and destained in ammonium bicarbonate (100 mM)/acetonitrile (45%) followed by in-gel digestion, which included reduction with Tris(carboxyethyl) phosphine (10 mM), alkylation with 50 mM iodoacetamide, and digestion with trypsin (mass spectrometry grade; Promega, Fitchburg, WI) or chymotrypsin (sequencing grade; Promega), and all steps were performed in 100 mM ammonium bicarbonate (pH 7.9). Extracted peptides were trapped by SPE C-Tip (Nikkyo Technos Co. Ltd., Tsukuba, Japan) (Nature protocols, 2, 1896) and eluted with 5 μL of 80% acetonitrile and 0.5% acetic acid, followed by direct injection into the matrix-assisted laser desorption ionization-time of flight (MALDI-TOF) mass spectrometer (MS) using a TOF/TOF 5800 system (AB Sciex, Framingham, MA). Eluted peptides were also analyzed by liquid chromatography/mass spectrometry (LC/MS) using electron-transfer/higher-energy collisional dissociation (EThcD). The peptides were separated using an UltiMate 3000 RSLCnano LC system (Thermo Fisher Scientific). A reversed-phase column (PepMap RSLC, C18, Thermo Fisher Scientific; 3μm, 0.075 × 150 mm) was used as the analytical column. The mobile phases were 0.1% (v/v) formic acid (buffer A) and acetonitrile containing 0.1% (v/v) formic acid (buffer B). The flow rate was set at 300 nL/min with a gradient of 2–40% buffer B for 90 min. The eluted peptides were automatically subjected to hybrid ion trap-Orbitrap mass spectrometry (Orbitrap fusion Lumos mass spectrometer, Thermo Fisher Scientific). The mass spectrometric conditions were as follows: electrospray voltage, 2.0 kV in positive ion mode; capillary temperature, 250°C; full mass resolution, 60 000; collision energy, 30% for data-dependent higher-energy collisional dissociation (HCD)-MS/MS experiment (Orbitrap resolution, 7500); full mass range, m/z 350–2000; MS/MS isolation width, 3.0 u (range of precursor ion ± 1.5). Electron transfer dissociation (ETD) and HCD (EThcD)-MS/MS data acquisition were triggered by the presence of glycan oxonium ions at m/z 138.0545 and 204.0867 in the HCD-MS/MS spectrum. Triggered EThcD-MS/MS was performed using an ion trap. Dynamic exclusion was set to exclude previously subjected precursor ions for 30 s. O-GlcNAc peptides in mouse Sox2 were identified by a database search using the search engine Bionic (ProteinMetrics, CA, USA) in Proteome Discoverer 2.0, software (Thermo Fisher Scientific). The search parameters were as follows: one missed cleavage, precursor ion mass tolerance of 10 ppm, mass tolerance of fragment ion in HCD-MS/MS spectra, 20 ppm, mass tolerance of fragment ion in EThcD-MS/MS spectra, 0.4 Da. Carboxyamidomethylation (57.021464 Da) was set as a static modification of Cys residues. Glycosylation of Ser and Thr residues with [HexNAc]_1_ (203.079 Da) was set as a dynamic modification. Targeted peptide spectrum matches (PSMs) were extracted based on a false discovery rate (FDR) <0.01 at the spectrum level.

### Expression of Sox2 in ES cells

For ES cells, transfection vectors were constructed using a pCAGIPuro vector with the Gateway cloning system (Invitrogen) according to the manufacturer’s instructions. Mouse ES cells were transfected with 4 μg of empty vector (EV), Sox2 wild-type (Sox2 WT), Sox2 FLAG-tag (FLAG-Sox2), and Sox2 FLAG-tag S263A (S263A Sox2) using Lipofectamine 2000 (Invitrogen). Site-directed mutagenesis of serine (Ser263) of Sox2 to alanine was performed using the KOD-plus-mutagenesis kit (Toyobo) according to the manufacturer’s instructions. Transfected cells were harvested 2 days post-transfection and used for immunoprecipitation, Western blotting analysis, and immunostaining using mouse anti-FLAG monoclonal Ab (M2) conjugated with horseradish peroxidase (F3165, Sigma-Aldrich), anti-O-GlcNAc Ab (RL2; sc-59624, Santa Cruz), and anti-O-GlcNAc Ab (CTD110.6; 9875, Cell Signaling).

### Statistical analyses

Levels of cell growth and Western blotted protein bands were expressed as means ±SE from three or more experiments. An unpaired Student’s t-test was used to compare the growth or intensity of the bands in the two experimental groups.

## Results

### Comparison of O-GlcNAcylated proteins detected by anti-O-GlcNAc antibodies RL2, CTD110.6, or wheat germ agglutinin

**Fig 1:**
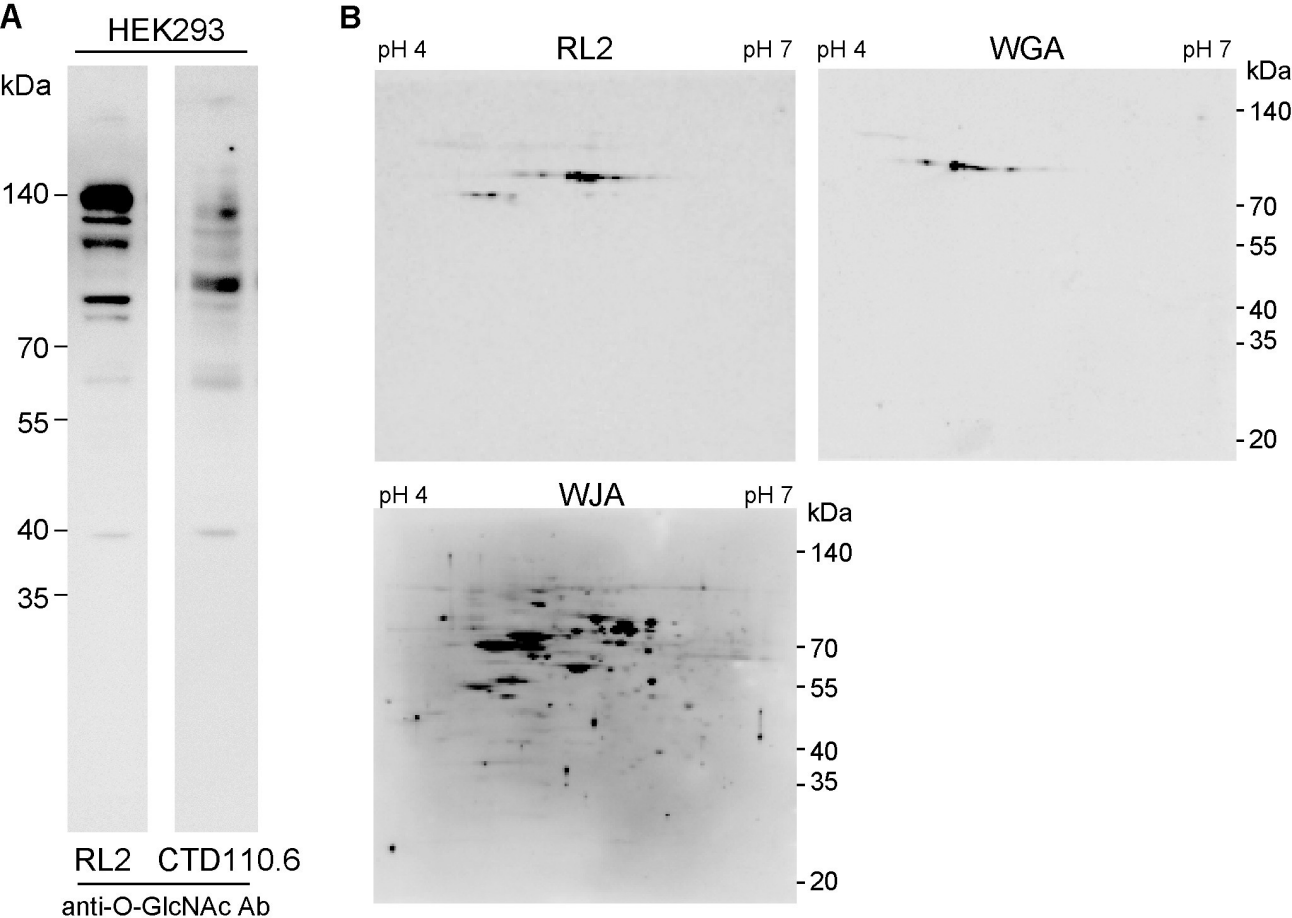
Different reactivity and sensitivity of O-GlcNAcylated proteins using conventional methods. (A) Different reactivity of O-GlcNAcylated proteins in HEK293 cells against monoclonal anti-O-GlcNAc antibodies, RL2 or CTD110.6. (B) Different sensitivity of O-GlcNAcylated proteins in HEK293 using anti-O-GlcNAc antibody, RL2, wheat germ agglutinin, and *Wisteria japonica* agglutinin (WJA, disaccharide-tag method).

**Fig 2:**
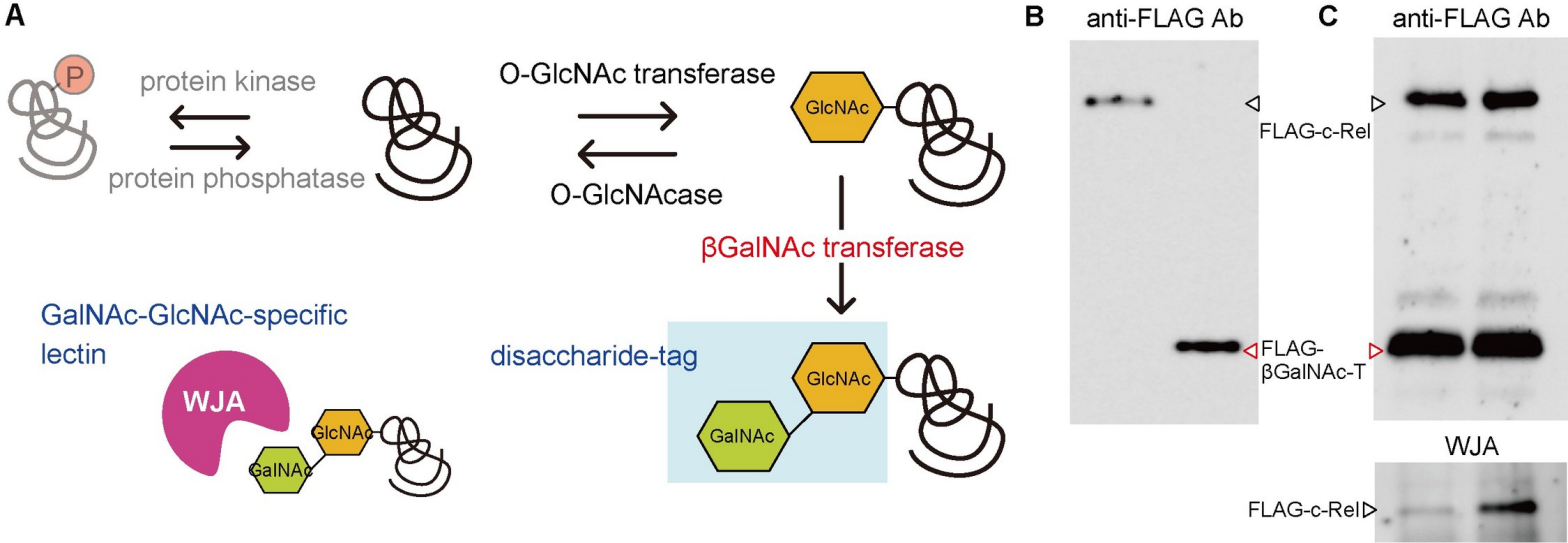
Schematic illustration of disaccharide-tag method to detect O-GlcNAcylated proteins. (A) Schematic illustration of disaccharide-tag method. (B) Western blotting of purified FLAG-c-Rel and FLAG-βGalNAc-T using anti-FLAG antibody. (C) Purified FLAG-c-Rel and FLAG-βGalNAc-T were mixed and incubated at 25°C for 12 h in the absence (left lane) or presence (right lane) of UDP-GalNAc. Amount of each protein was confirmed by Western blotting using anti-FLAG antibody and extension of O-GlcNAc residue was confirmed by Western blotting using WJA.

O-GlcNAcylated proteins from HEK293 cells were analyzed by SDS-polyacrylamide gel electrophoresis (SDS-PAGE) followed by Western blotting using anti-O-GlcNAc antibodies RL2 or CTD110.6, which are widely used for the detection of O-GlcNAcylated proteins. Although the same HEK293 cell lysate was used as a sample, the antibody staining patterns were different when anti-O-GlcNAc Ab RL2 or CTD110.6 was used (Fig 1A). Different reactivities may depend on the amino acid sequence surrounding O-linked N-acetylglucosaminylated Ser or Thr because these monoclonal antibodies recognize not only O-GlcNAc residues but also sugar-modified peptide moieties. To overcome the different reactivity of O-GlcNAcylated proteins against anti-O-GlcNAc Abs, we tried to establish different detection methods for O-GlcNAcylated proteins. In this experiment, we expressed soluble β4GalNAc transferase in cytoplasm by removing both its signal sequence and transmembrane domain and the nucleic and cytoplasmic proteins of the cell to bring O-GlcNAc residue to GalNAcβ1-4GlcNAc disaccharide, which we named "disaccharide-tag" method (Fig 2A). In this method, we used *Wisteria japonica* agglutinin (WJA) as a probe to detect GalNAcβ1-4GlcNAc disaccharide, because WJA is highly specific for GalNAcβ1-4GlcNAc, with a dissociation constant (K_d_) of 7.1 x 10^−6^ M, as described previously [8].

HEK293 cell lysates were analyzed by two-dimensional gel electrophoresis (2D-PAGE) followed by Western blotting using anti-O-GlcNAc antibody RL2 or wheat germ agglutinin. Simultaneously, HEK293 cells transfected with pFLAG-CMV3-β4GalNAc-TA were analyzed by 2D-PAGE followed by Western blotting using WJA lectin. Fig 1B shows the results of detection of O-GlcNAcylated proteins from HEK293 cells using conventional anti-O-GlcNAc Ab RL2, wheat germ agglutinin, or our distinct disaccharide-tag method. In the case of anti-O-GlcNAc Ab detection, N,N’-GlcNAc_2_-modified proteins reacted with this antibody, as reported previously [13,14]. When wheat germ agglutinin was used as a probe, fewer spots were detected. Furthermore, wheat germ agglutinin binds to N-acetyl groups of both N-acetylglucosamine and N-acetylneuraminic acid, some of which may be membrane proteins or secreted proteins with N-glycans or O-glycans. In contrast, Western blotting using WJA lectin in combination with soluble β4GalNAc-TA in HEK293 cells detected many spots with high sensitivity (Fig 1B).

We next tried to confirm whether soluble β4GalNAc transferase can transfer GalNAc to the GlcNAc residue of the O-GlcNAcylated proteins in an *in vitro* experimental system. FLAG-tagged soluble β4GalNAc transferase and the human c-Rel protein, known to have O-GlcNAc modification [15], were expressed in HEK293 cells. After immunoprecipitation from each cell lysate using an anti-FLAG antibody and recovery with a FLAG peptide, purification of each protein was confirmed by SDS-PAGE followed by Western blotting with an anti-FLAG antibody. Purification of each soluble β4GalNAc transferase and c-Rel protein was confirmed (Fig 2B). Both purified proteins were then mixed and incubated at 25°C for 12 h in the presence or absence of UDP-GalNAc. When the reaction solution was subjected to SDS-PAGE, blotted onto the membrane, and stained with anti-FLAG antibody and WJA lectin, the c-Rel protein band was strongly stained by WJA only in the presence of UDP-GalNAc (Fig 2C). These results indicate that soluble β4GalNAc transferase can act on GlcNAc residues of GlcNAcylated proteins to elongate GlcNAc to GalNAcβ1-4GlcNAc.

### A modified disaccharide-tag method using a β4GalNAc transferase with a nuclear localization signal

**Fig 3:**
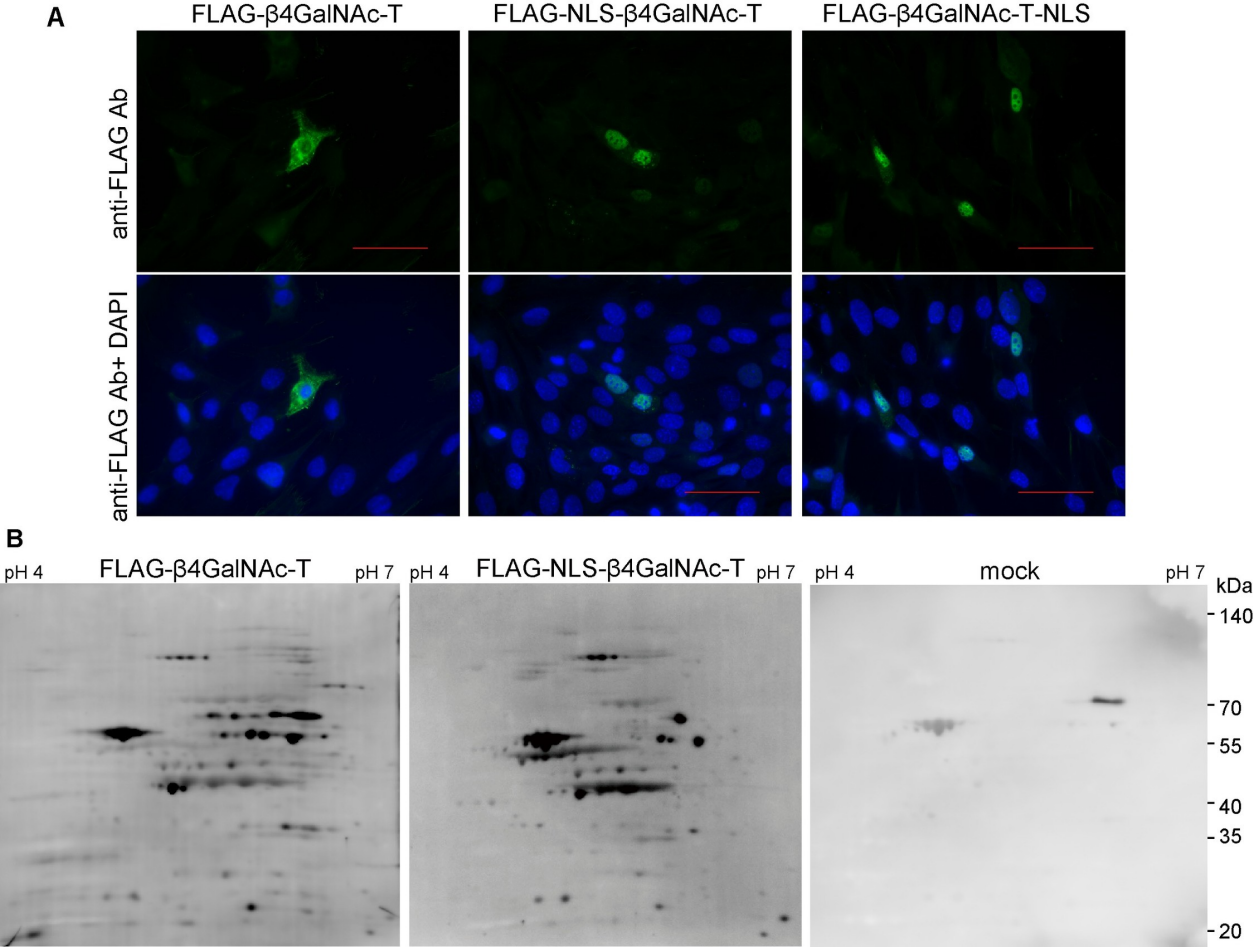
A modified disaccharide-tag method using a β4GalNAc transferase with a nuclear localization signal. (A) Expression of FLAG-tagged soluble β4GalNAc transferase with or without nuclear localization signal (NLS). HEK293 cells expressing FLAG-tagged soluble βGalNAc (FLAG-βGalNAcT), FLAG-tagged soluble βGalNAc with NLS at N-terminus (FLAG-NLS-βGalNAcT), or FLAG-tagged soluble βGalNAc with NLS at C-terminus (FLAG-NLS-βGalNAcT) were stained with Alexa488-labeled anti-FLAG Ab and DAPI. White bar indicates 10 nm. (B) O-GlcNAcylated proteins in mouse pancreatic ductal adenocarcinoma cells (YamaPaca-6) transfected with pFLAG-CMV3-NLS-β4GalNAc-TA, pFLAG-CMV3-β4GalNAc-TA, or pFLAG-CMV3 (mock), respectively, were detected by disaccharide-tag method.

To detect O-GlcNAc modification of proteins, especially in the nucleus of the cell, we constructed expression vectors encoding soluble β4GalNAc-TA with a nuclear localization signal (NLS) from the SV40 large T antigen [16] at the N- or C-terminus, respectively (pFLAG-CMV3-NLS-β4GalNAc-TA or pFLAG-CMV3-β4GalNAc-TA-NLS). After the plasmids were transfected into HEK293 cells, localization of β4GalNAc-TAs with NLS at the N- or C-terminus in the cells was examined by immunostaining using anti-FLAG Ab of the transfected cells. Soluble FLAG-β4GalNAc-TA was distributed mainly in the cytoplasm (Fig 3A left). In contrast, soluble FLAG-β4GalNAc-TAs with NLS at the N- or C-terminus localized specifically in the nucleus, as expected (Fig 3A middle and right). Using these soluble FLAG-β4GalNAc-TAs with different cellular localizations, the staining patterns of O-GlcNAcylated proteins were different from each other. We then analyzed O-GlcNAcylated proteins from mouse pancreatic ductal adenocarcinoma cells using the above two kinds of soluble FLAG-β4GalNAc-TA with or without NLS (Fig 3B). Although the same amount of protein samples were analyzed by 2D-PAGE followed by Western blotting using WJA, O-GlcNAcylation of proteins appeared to be increased in the nucleus. However, some spots of O-GlcNAcylated proteins were rich in the cytoplasm, indicating that the subcellular localization of O-GlcNAcylated proteins may depend on their functions in the cell.

### Identification of O-GlcNAcylated proteins associated with epiblast-like stem cell differentiation using mouse ES cells

**Fig 4:**
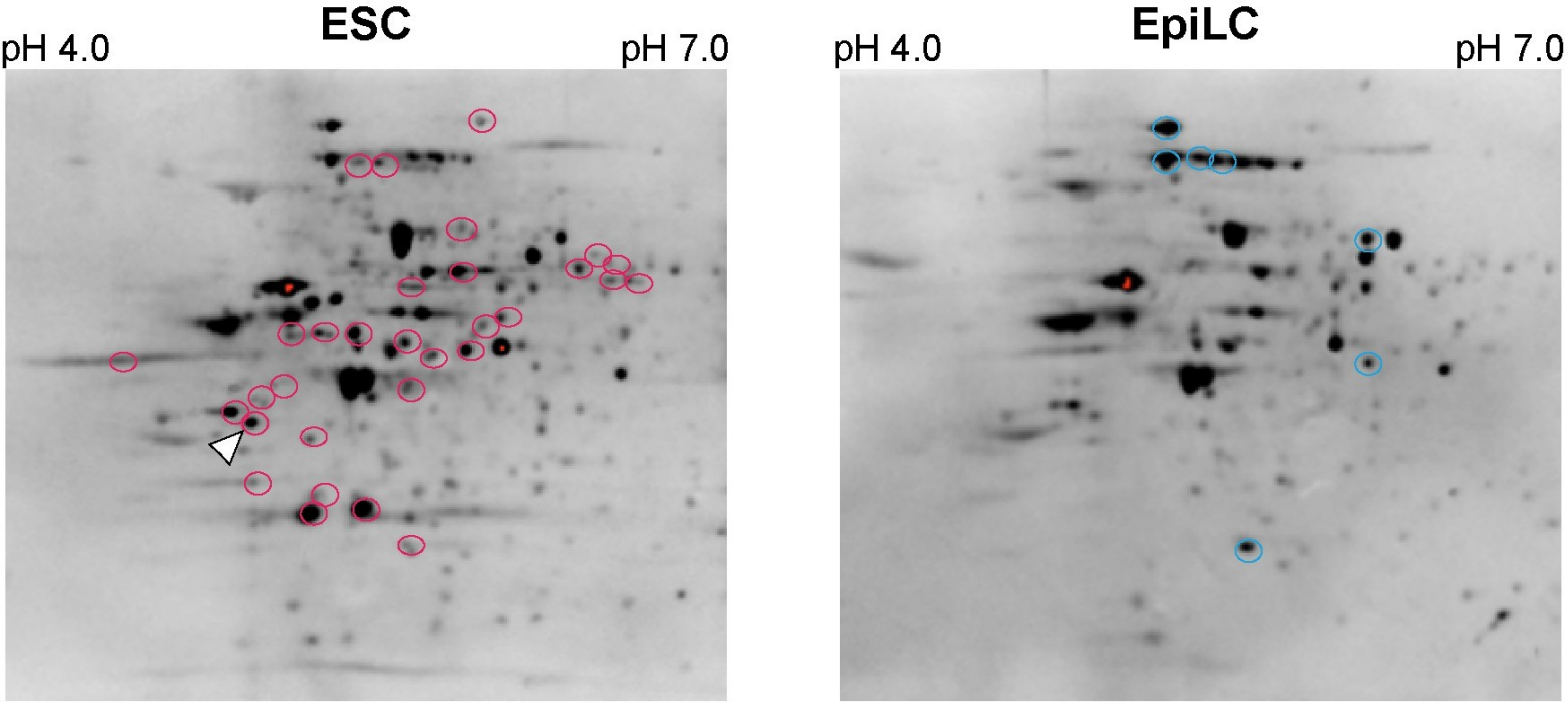
Detection of O-GlcNAcylated proteins from ESC or EpiLC using disaccharide-method. Increased spots in ESCs are indicated in red circles and increased spots in EpiLCs are indicated in blue circles. The spot of Sox2 is shown in white arrowhead in ESCs.

**Fig 5:**
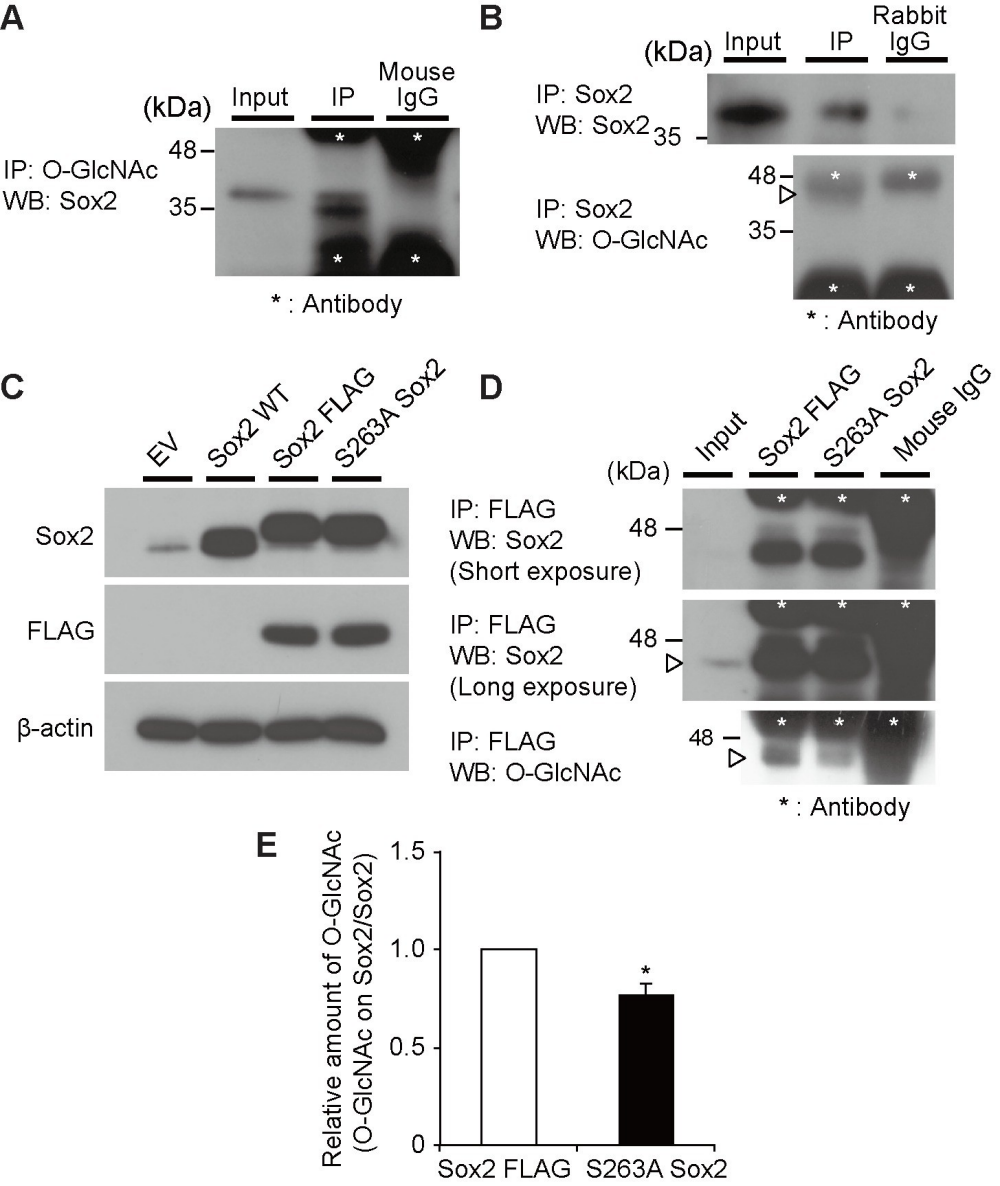
Sox2 is O-GlcNAcylated on S263 in mouse ESCs. (A) Representative image of a Western blotting using an anti-Sox2 Ab on the immunoprecipitated fraction (IP) precipitated with an antibody against O-GlcNAc (RL2). The input represents the total mouse ES cell lysate. Similar results were obtained in three independent experiments. (B) Representative image of a Western blotting using an anti-Sox2 Ab, anti-OGT Ab, and anti-O-GlcNAc Ab (RL2) on the immunoprecipitated fraction (IP) precipitated with an antibody against Sox2. The input represents the total ES cell lysate. The arrowhead indicates the Sox2 protein. Similar results were obtained in three independent experiments. (C) Representative image of Western blotting using an anti-Sox2 Ab, anti-FLAG Ab, and anti-β-actin Ab, in mES cells at 2 days after transfection with an empty vector (EV), Sox2 wild-type (Sox2 WT), Sox2 FLAG-tag (Sox2 FLAG), and Sox2 FLAG-tag S263A (S263A Sox2). Similar results were obtained in three independent experiments. (D) Representative image of immunofluorescence using anti-Sox2 Ab, in ES cells at 2 days after transfection with an empty vector (EV), Sox2 wild-type (Sox2 WT), Sox2 FLAG-tag (Sox2 FLAG), and Sox2 FLAG-tag S263A (S263A Sox2). (E) Band intensity of O-GlcNAc on Sox2 in (D) was normalized against total Sox2 and shown as fold change relative to Sox2 FLAG cells. The values are shown as means ± SEM of three independent experiments. Significant values are indicated as *p < 0.05.

Next, the disaccharide-tag method was applied to mouse ESCs, and the changes in O-GlcNAcylation of proteins accompanied by differentiation to epiblast-like cell (EpiLC) were examined. We transfected pFLAG-CMV3-NLS-β4GalNAc-TA into mouse ESCs or EpiLCs, and prepared cell lysates. These samples were subjected to 2D-PAGE, Western blotting, and WJA staining. The same cell lysates were subjected to 2D-PAGE under the same conditions and proteins were visualized by silver staining. Thirty-one spots (Fig 4, red circles) were increased in ESCs, whereas seven spots were increased in EpiLCs (Fig 4, blue circles). To identify the increased O-GlcNAcylated proteins in the ESCs compared with EpiLCs, their corresponding spots were excised from the same gel, followed by gel digestion with trypsin. The obtained peptide fragments were analyzed using matrix-assisted laser desorption ionization-time of flight mass spectrometry (MALDI-TOF MS/MS) and liquid chromatography tandem mass spectrometry (LC-MS/MS) and several proteins were identified. Among these proteins, we focused on the transcription factor Sox2 because this protein was reported to be involved in the self-renewal of ESCs, and its O-GlcNAcylation was also discussed [17,18]. We then tried to identify O-GlcNAcylated sites on their tryptic fragments and successfully identified an O-GlcNAcylated site on Sox2. The tryptic fragment from mouse Sox2 corresponding to residues from Ser248 to Arg264 was found to be O-GlcNAcylated at Ser263 (S1 Fig). The residue Ser263 was modified with HexNAc but not HexNAc-HexNAc, indicating that a part of the O-GlcNAc was elongated to GalNAc-GlcNAc by β-GalNAc-T; however, it was sufficient to detect O-GlcNAcylated proteins by WJA staining. The lysates from the ESCs were subjected to immunoprecipitation with anti-O-GlcNAc (RL2, Fig 5A) or anti-Sox2 Abs (Fig 5B). Sox2 protein was O-GlcNAcylated because immunoprecipitation using anti-O-GlcNAc Ab was stained with anti-Sox2 Ab and *vice versa* (Fig 5A and 5B) To confirm that Ser263 is a novel O-GlcNAcylation site on Sox2 in mouse ES cells, we prepared expression plasmids encoding wild-type Sox2, FLAG-tagged Sox2, or its S263A mutant and then transfected them into mouse ES cells. Expression of mouse Sox2, FLAG-tagged Sox2, or FLAG-tagged Sox2(S263A) mutant was confirmed (Fig 5C). The precipitates using anti-FLAG antibody from lysates expressing wild-type FLAG-tagged Sox2 or its S263A mutant expressing cells were subjected to polyacrylamide gel electrophoresis followed by Western blotting using anti-Sox2 Ab or anti-O-GlcNAc Ab, respectively (Fig 5D). Although FLAG-tagged Sox2 or its S263A mutant were equally precipitated (Fig 5D upper and middle panels), wild-type FLAG-tagged Sox2 was strongly stained with anti-O-GlcNAc antibody compared to its S263A mutant (Fig 5D, lower panel). Furthermore, the S263A mutant of Sox2 was stained with anti-O-GlcNAc Ab, RL2, slightly (approximately 80%, Fig 5E), indicating that Ser263 of Sox2 was one of the main O-GlcNAcylated residues, but other residues may also be modified with GlcNAc.

## Discussion

In this study, we established a disaccharide-tag method, which was a highly sensitive and novel method for detecting O-GlcNAcylated proteins. This method is much more sensitive than detection by conventional anti-O-GlcNAc antibodies or wheat germ agglutinin, and we could comprehensively compare O-GlcNAcylated proteins using cultured cells under various conditions. It was also demonstrated that not only was the detection sensitivity high, but it also has additional excellent features, as discussed below.

N- and O-glycans are modifications to all secretory or membrane-bound proteins and are constantly changing their structures in response to various conditions within the cell. In contrast, O-GlcNAc modification is a glycosylation of a protein that occurs in the cytoplasm or nucleus, and its modification is reversible [2,3]. Therefore, O-GlcNAcylated proteins are not the largest component of all proteins present and are therefore difficult to detect. Because of this difficulty in detection, analysis of the biological significance of O-GlcNAc modification remains unresolved. Therefore, an experimental system that sensitively detects and compares this modification is required before the significance of this modification can be determined.

A detection method using an anti-O-GlcNAc antibody has often been used to detect O-GlcNAcylated proteins. However, since the amino acid sequence surrounding the O-GlcNAc modification site is also included in the recognition epitope of the antibody, the sensitivity of detection differs depending on the antibody used (Fig 1A). Consequently, a comparative analysis was undertaken with a large bias, and it was considered unsuitable for the quantitative analysis of O-GlcNAcylated proteins. In contrast, the disaccharide-tag method is based on a simple binding between GalNAc-GlcNAc and WJA lectin, and it does not depend on the amino acid sequence surrounding the O-GlcNAc-modified residue. Therefore, it was considered excellent in that it was possible to intuitively make a quantitative comparison of.

The disaccharide-tag method is superior in that it has a high sensitivity. There is a detection method using WGA lectin as one of the methods for detecting O-GlcNAc modified proteins [19]. Because the WGA lectin used in this method binds not only to GlcNAc residues but also to N-acetylneuraminic acid [5], this method detects not only O-GlcNAc-modified proteins present in the cytoplasm and nucleus, but also sialic acid-containing glycoproteins in the cell lysate. Because N-acetylneuraminic acid binds strongly to WGA through ionic interaction, succinylation of WGA abrogates the binding to N-acetylneuraminic acid but not to GlcNAc [6]. Therefore, succinylated WGA is also used to detect O-GlcNAcylated proteins specifically [7]. The binding force between WGA and GlcNAc is as weak as K_a_ = 1.3 x 10^3^ M^-1^ [20], which is considered to be the reason for its poor sensitivity (Fig 1B). In contrast, binding between GalNAc-GlcNAc used in the disaccharide-tag method and WJA lectin has a much stronger K_a_ of 1.4 x 10^5^ M^-1^ which is why detection sensitivity is high in the disaccharide-tag method [8]. In addition, sugar chains with the GalNAc-GlcNAc sequence were hardly expressed in animal cells. The GalNAc-GlcNAc structure is found at the non-reducing end of the N-type sugar chain of pituitary glycoprotein hormones (leutropin, thyrotropin, follitropin), which serves as a clearance signal from the blood stream [21]. These glycoprotein hormones are present only in the pituitary gland and are not expressed in other tissues and cells. In addition, because GalNAc-GlcNAc has a strong affinity for WJA lectin, it is possible to selectively purify only proteins with GalNAc-GlcNAc by affinity chromatography using a WJA-immobilized column. Alternatively, β1,4-galactosyltransferase from bovine milk is also used to detect O-GlcNAc modification of proteins. In this experiment, ^3^H-labeled UDP-Gal is used as a substrate to elongate O-GlcNAc residue to Galβ1-4GlcNAc. However, UDP-Gal is frequently converted into other UDP-sugars in the cell, so other sugar chains may be also labeled with tritium. Furthermore Galβ1-4GlcNAc structure produced by the action of β1,4-galactosyltransferase is widely found in complex-type N-glycans. Because of such reason, glycoproteins without O-GlcNAc modification may be also detected and purified together. To increase the sensitivity of O-GlcNAcylated proteins, mutated β1,4-galactosyltransferase and azido-modified GalNAz as a substrate are used in a Click-iT O-GlcNAc enzymatic labeling system (Invitrogen) [7]. This system can be detected in a relatively short period, however there is a risk that glycoproteins other than O-GlcNAc modified proteins will be also detected because GalNAz is metabolized to GlcNAz and so on. To overcome such weakness, disaccharide-tag method using soluble GalNAc transferase may be suitable for the specific detection and purification of O-GlcNAcylated proteins. We think that the weak point of our disaccharide-tag method, including above Click-iT O-GlcNAc method, is to require the expression of glycosyltransferases in cells. Therefore these methods cannot be applied to pathological specimen and in this point of view another method needs to be developed in future.

When we compared O-GlcNAc modified proteins using ES and EpiLC cells, we found that the O-GlcNAc modification of transcription factor Sox2 was decreased and this was accompanied by differentiation to EpiLCs. In a comprehensive and comparative analysis using a probe for O-GlcNAc modification, it was not possible to determine whether this modification was enhanced or whether the expression of the protein itself was increased. Consequently, it was necessary to examine each identified protein in detail. After immunoprecipitation of this protein with an anti-Sox2 antibody, one of the O-GlcNAc modification sites on Sox2 was found to be Ser263 by ETdhcD LC-MS/MS. However, this modification was not the disaccharide of GalNAc-GlcNAc, but GlcNAc itself (S1 Fig). Since the Sox2 protein was detected by WJA on two-dimensional gel electrophoresis, it is considered that at least a part of the O-GlcNAc residues of this protein was extended to GalNAc-GlcNAc. In this analysis, cell lysates were subjected to two-dimensional electrophoresis, and spots stained with WJA were excised to identify proteins. However, the efficiency of transformation of HEK293 cells with Lipofectamine 2000 was approximately 10%, and GalNAc added by GalNAc transferase is neutral and has a small molecular weight. Thus, it is likely that the molecular weight and isoelectric point of GalNAc-modified Sox2 protein can migrated to the same position as unmodified ones, and the O-GlcNAc modification on Ser263 was identified from the major unmodified Sox2 protein. Another possibility was that O-GlcNAc modification occurred at multiple sites of the Sox2 protein, and GlcNAc of Ser263 did not extend and remained unaltered, although GlcNAc of another residue became GalNAc-GlcNAc. Alternatively, both possibilities may have occurred simultaneously.

The biological mechanism by which O-GlcNAc modification is reversible remains poorly understood, but it is expected to be essential for cell survival and protein function. If all O-GlcNAc modifications were extended to GalNAc-GlcNAc, it could threaten cell survival. Taking this point into consideration, the time course after the expression of GalNAc transferase in the cytoplasm was taken and the survival rate of the GalNAc transferase-expressing cells was continuously measured. The data showed that survival rate of GalNAc transferase-expressing cells was not different from the cells without expression. Because elongation of the O-GlcNAc occurred only in a part, it had no adverse effect on soluble GalNAc transferase-expressing cells. To elucidate the biological significance of O-GlcNAc modification, it is necessary to distinguish between those with O-GlcNAc modification and those without O-GlcNAc modification at a specific residue of a protein. For this purpose, an antibody specific to the peptide sequence containing the O-GlcNAc modification is required to investigate the intracellular localization of O-GlcNAcylated proteins, or immunoprecipitation could be performed to pull down the interacting proteins. Therefore, techniques for the identification of O-GlcNAcylation at specific sites are considered to represent a powerful approach. To produce such an antibody, it is essential to establish a method for identifying the O-GlcNAc-modified protein and its modification sites with high sensitivity. In conclusion, our method is excellent because it represents the most sensitive approach and will be a powerful approach for the identification of O-GlcNAc-modified proteins and the biological significance of O-GlcNAc modification.

## Supporting information

S1 Fig ETD-MS/MS spectra of mouse Sox2 tryptic peptide. MS/MS spectra of a mouse Sox2 tryptic peptide showed the presence of HexNAc modification. The MASCOT program showed that Ser16 of the peptide was most likely modified with HexNAc.
(TIF)

S1 Raw images (PDF)
