# Supplementary figures and images for: Disaccharide-tag for highly sensitive identification of O-GlcNAc-modified proteins in mammalian cells
Source: PLoS One. 2022 May 23;17(5):e0267804. doi: 10.1371/journal.pone.0267804 (PMC9126400; doi:10.1371/journal.pone.0267804)

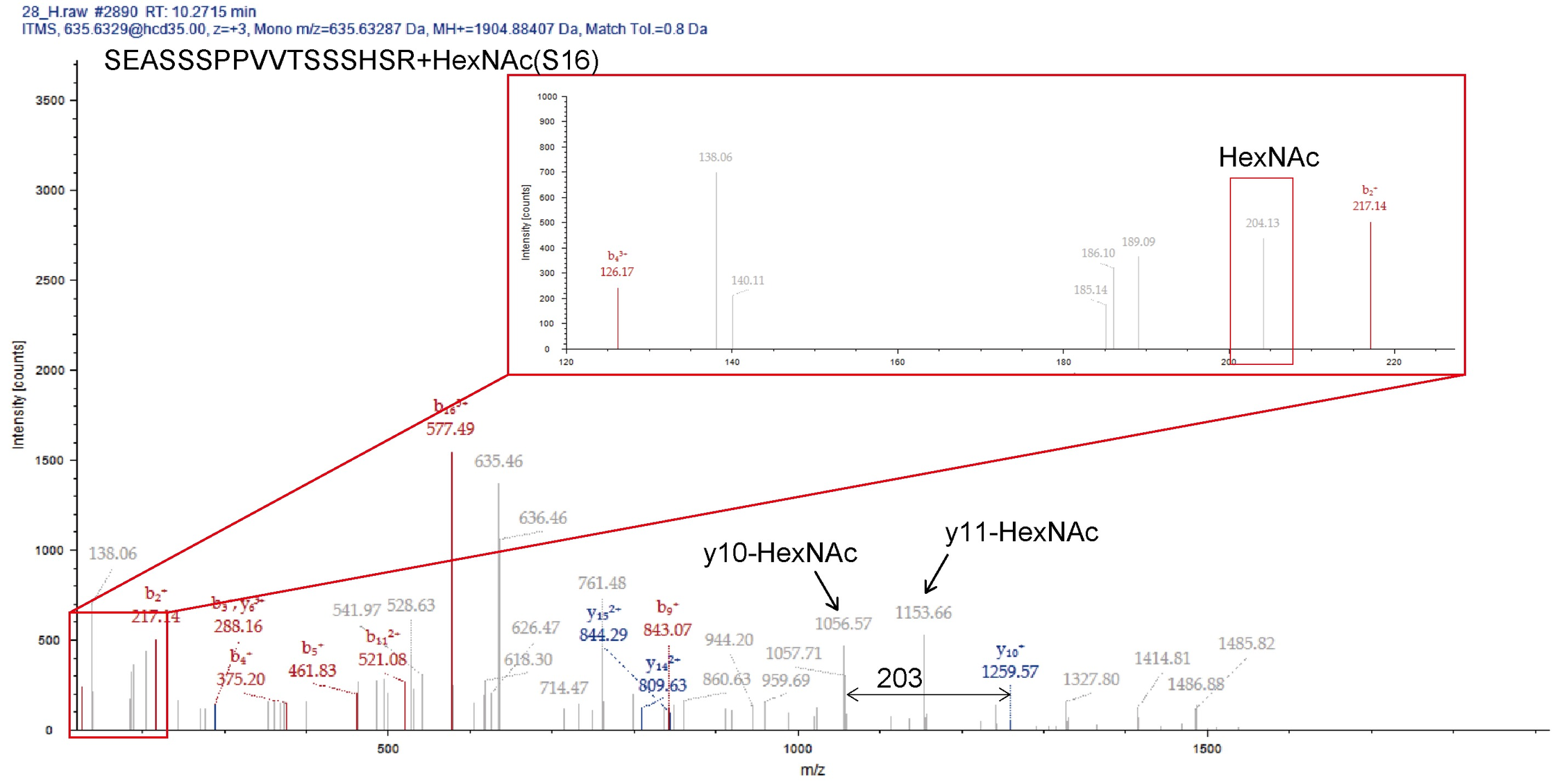

Supplement: S1 Fig — MS/MS spectra of a mouse Sox2 tryptic peptide showed the presence of HexNAc modification. The MASCOT program showed that Ser16 of the peptide was most likely modified with HexNAc. (TIF) [file pone.0267804.s001.tif]
