# Supplementary material for: Disaccharide-tag for highly sensitive identification of O-GlcNAc-modified proteins in mammalian cells
Source: PLoS One. 2022 May 23;17(5):e0267804. doi: 10.1371/journal.pone.0267804 (PMC9126400; doi:10.1371/journal.pone.0267804)

All images were detected using Image Quant LAS4000 (GE healthcare) and ECL Western blotting detection reagent (GE Healthcare) as a substrate.

Fig 1A left

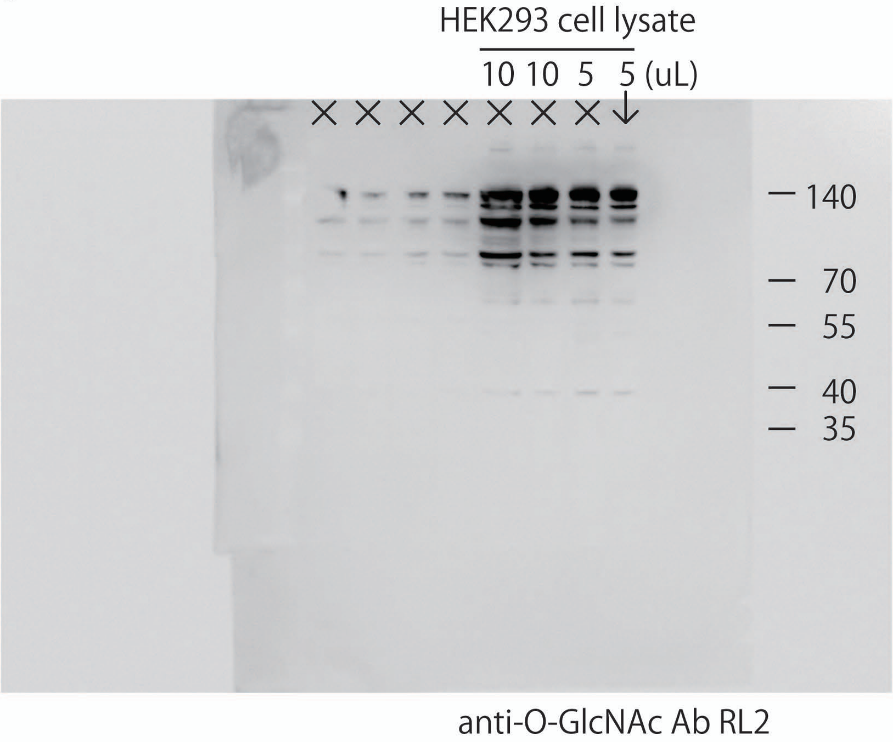

Fig 1A right

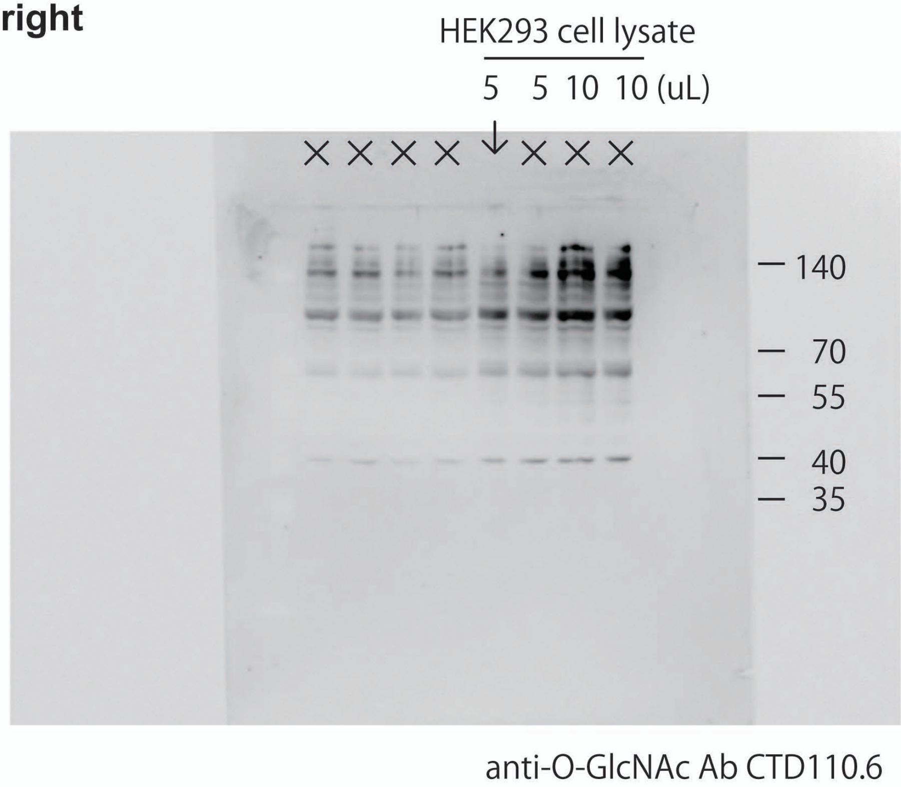

Fig 1B upper left

anti-O-GlcNAc Ab RL2

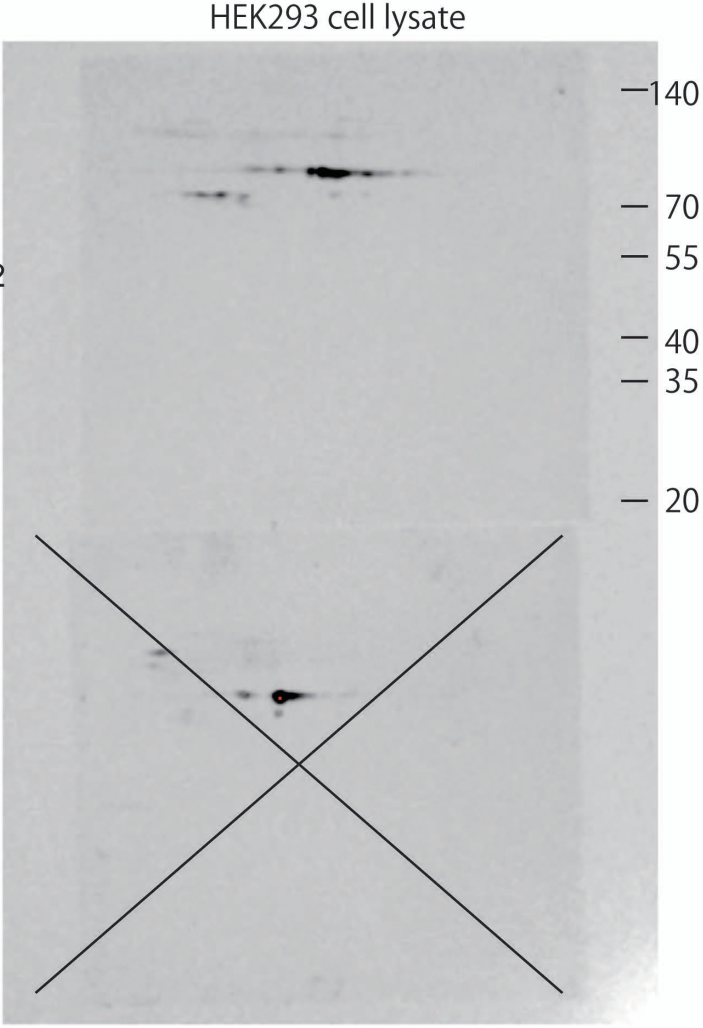

Fig 1B upper right

wheat germ agglutinin
(WGA)

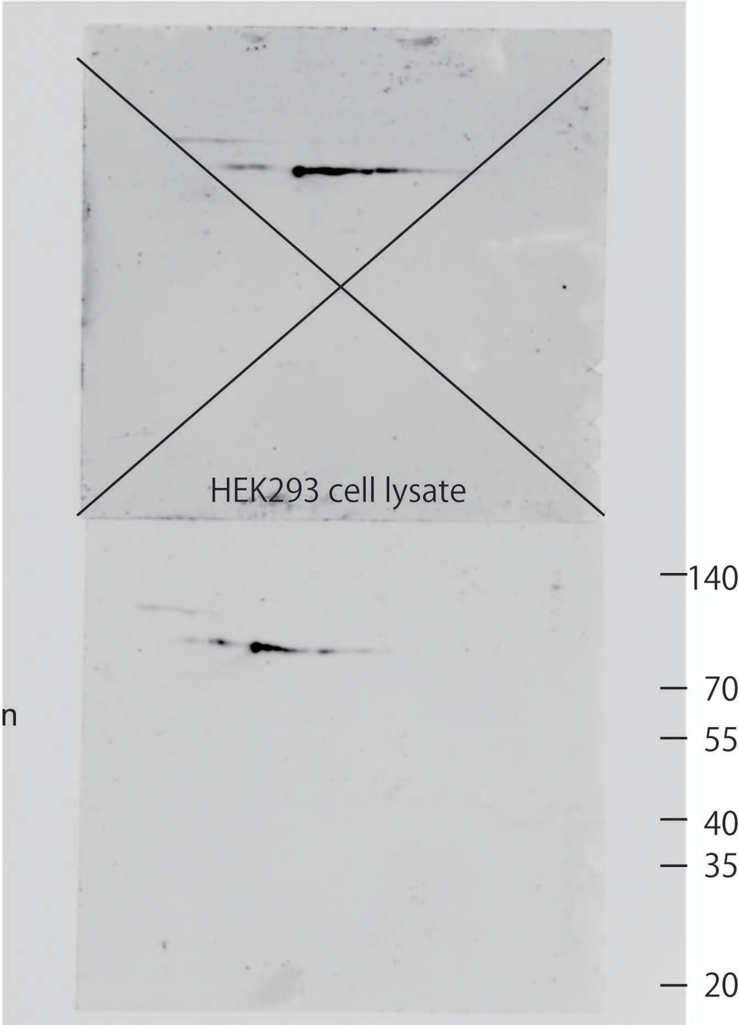

Fig 1B lower left

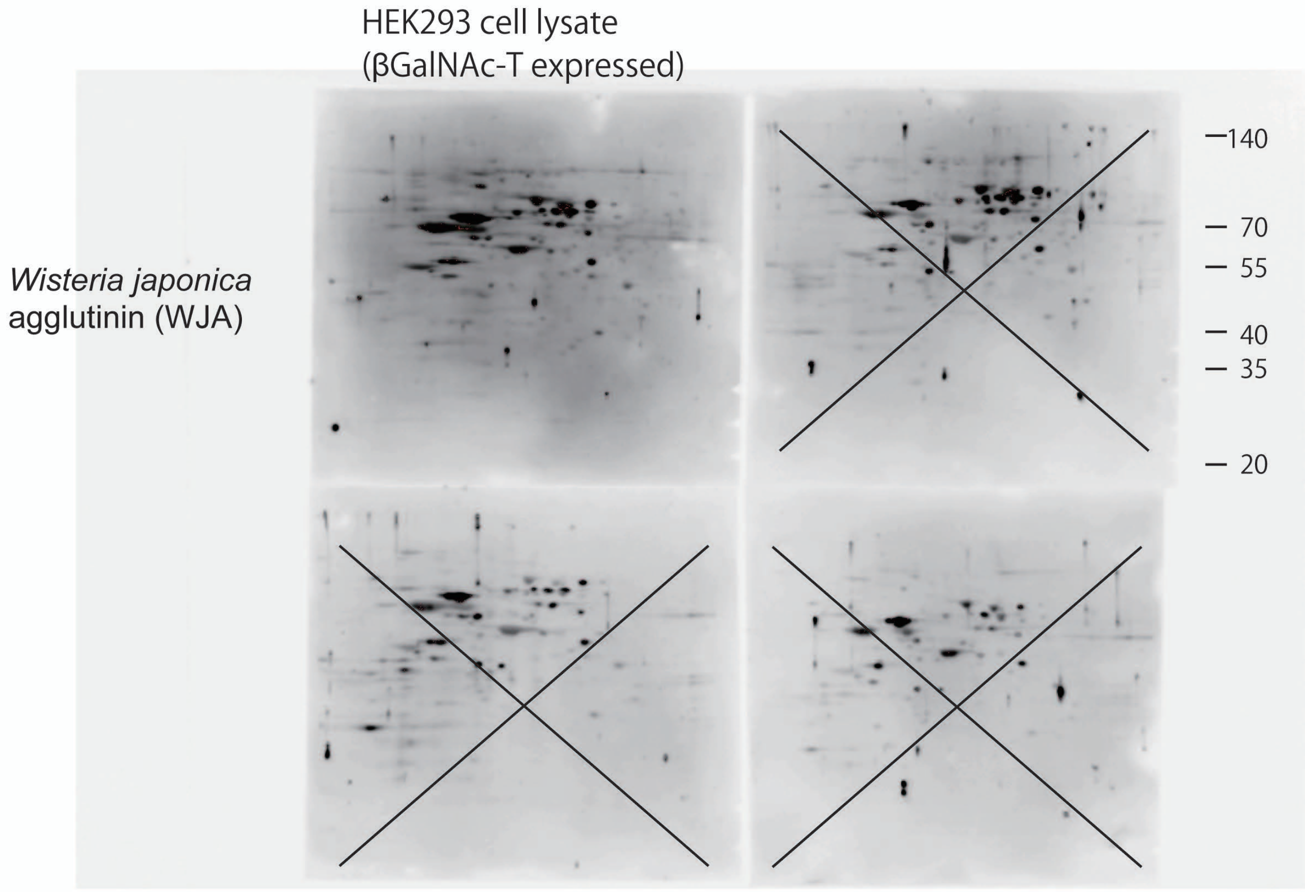

Fig 2B

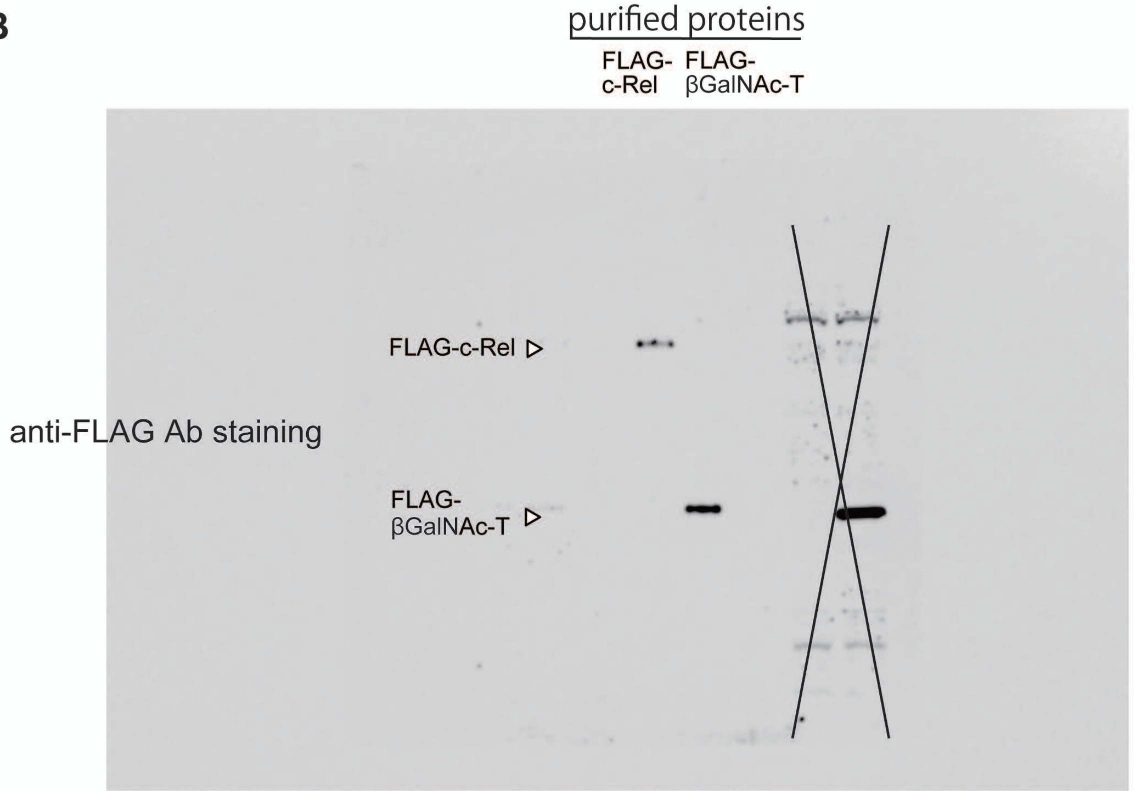

Fig 2C upper

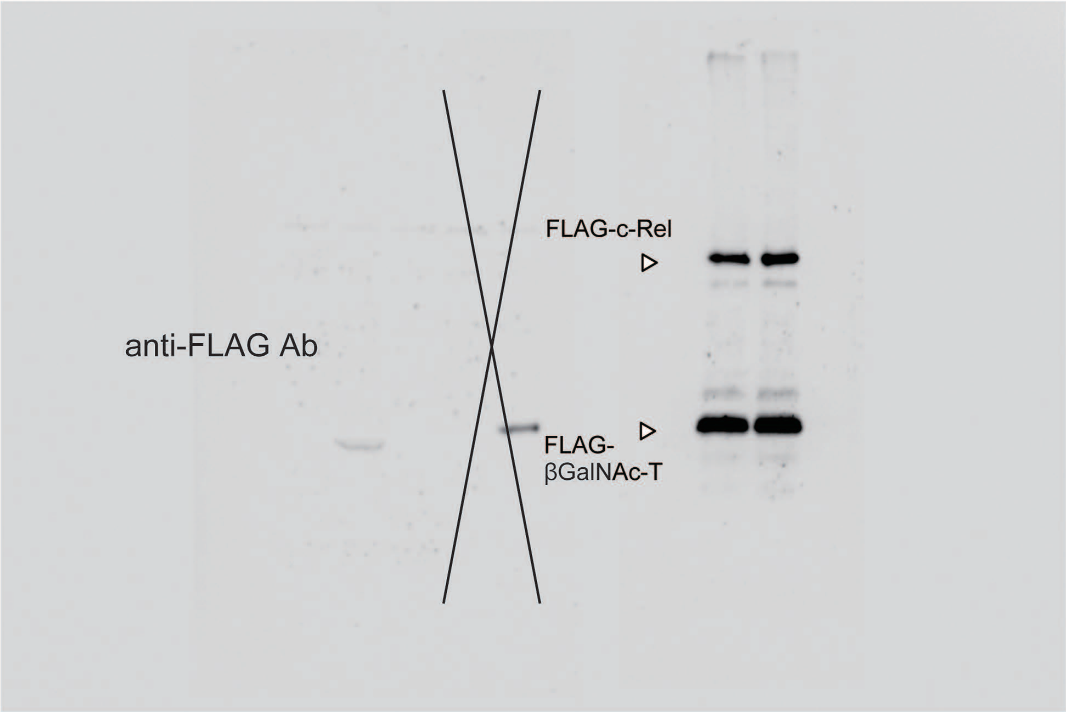

Fig 2C lower

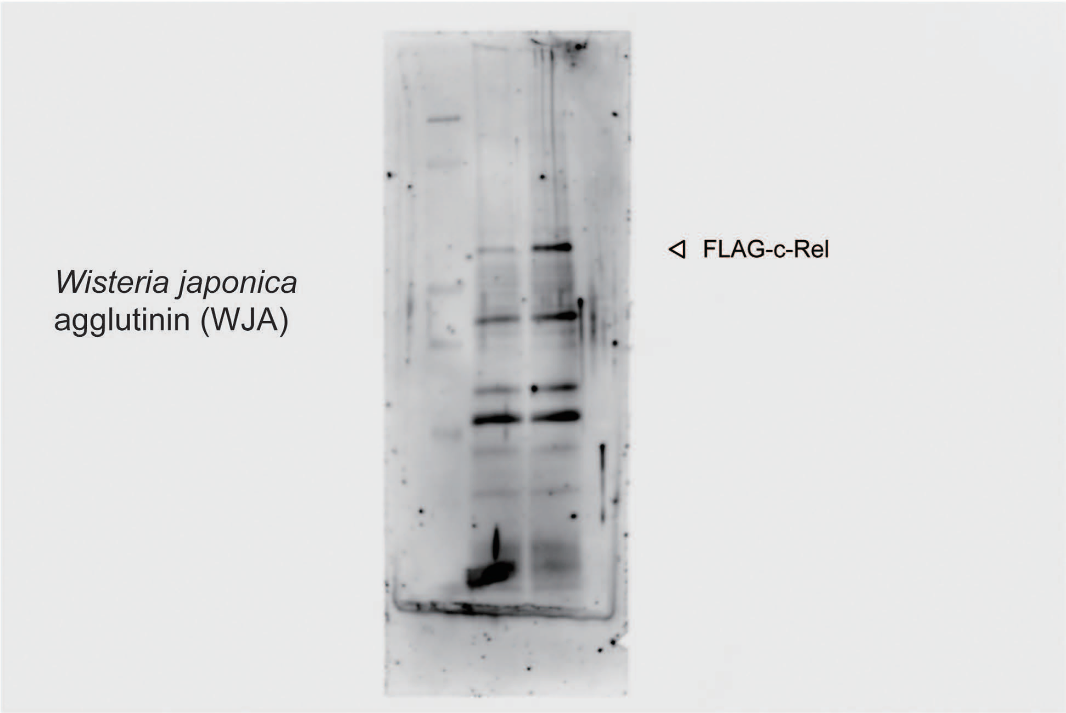

Fig 3 *Wisteria japonica* agglutinin (WJA) staining

Fig 3B middle

FLAG-NLS-β4GalNAc-T
-transfected HEK293 cell

Fig 3B left

FLAG-β4GalNAc-T
-transfected HEK293 cell

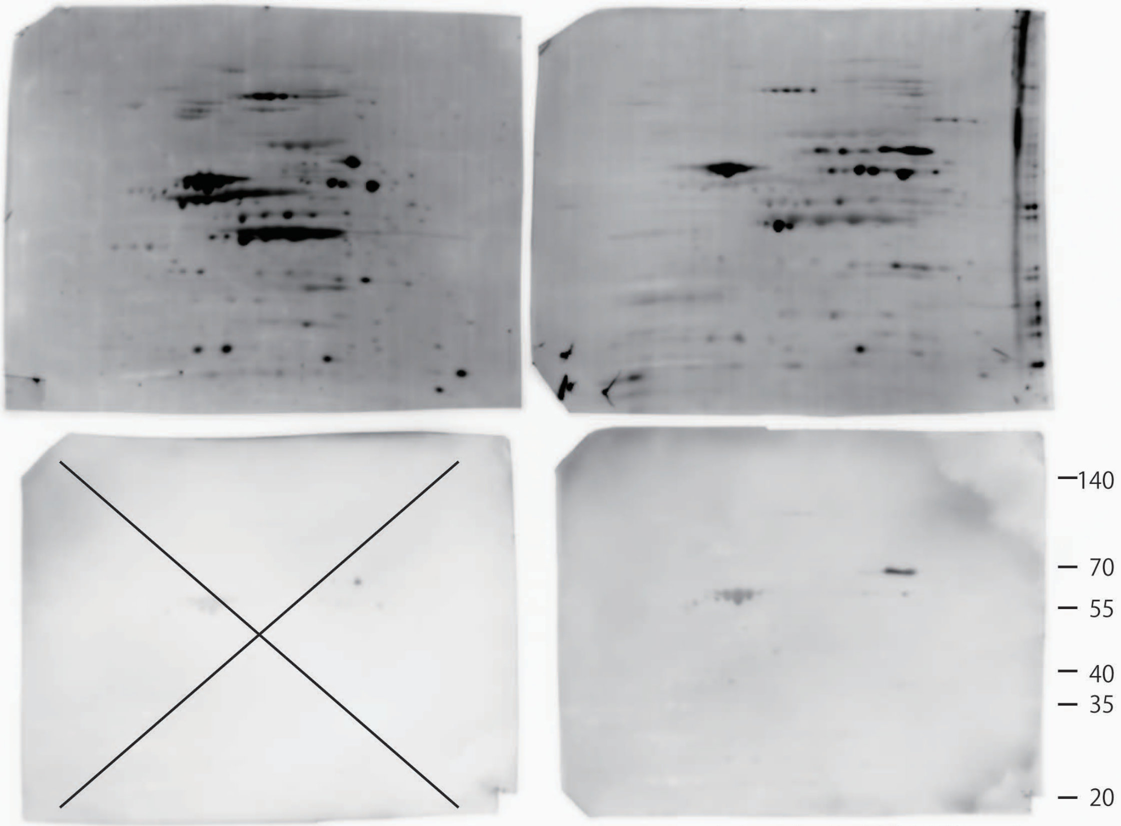

mock-transfected HEK293 cell

Fig 3B right

Fig 4 *Wisteria japonica* agglutinin (WJA) staining

Fig 4 left embryonic stem cell

Fig 4 right epiblast-like stem cell

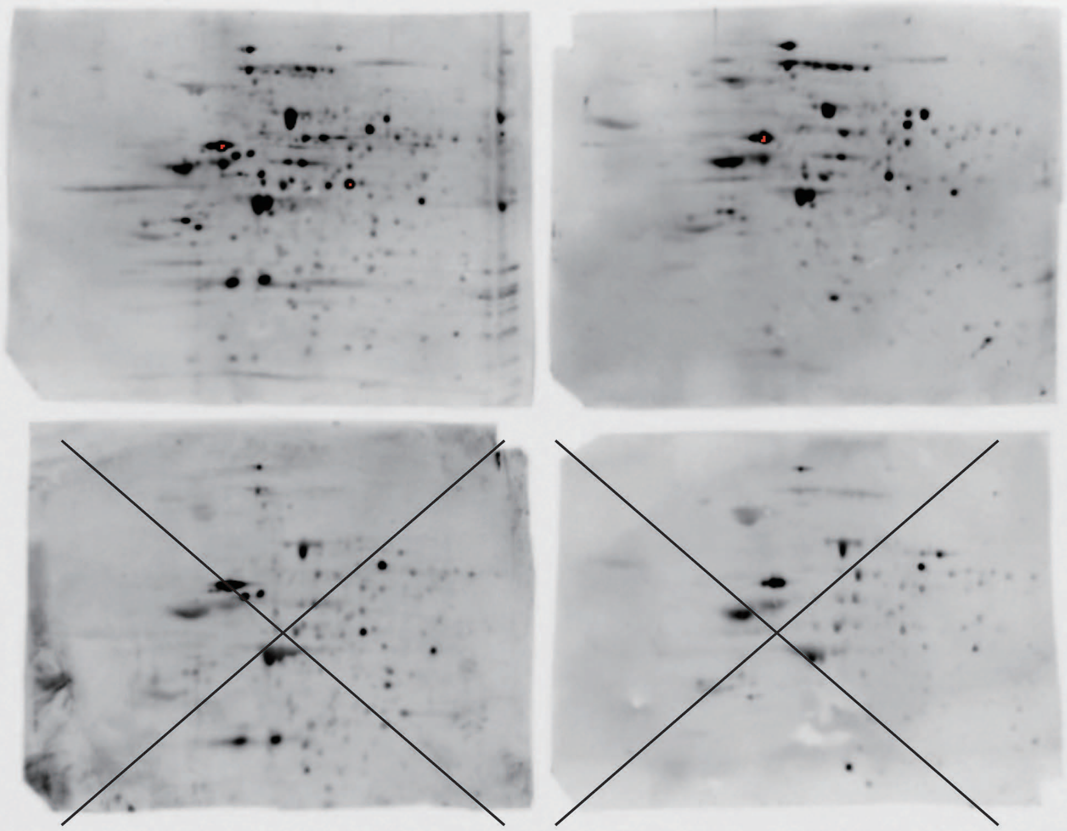

Fig 5

Fig 5A

IP: O-GlcNAc Ab
(RL-2)
WB: Sox2 Ab

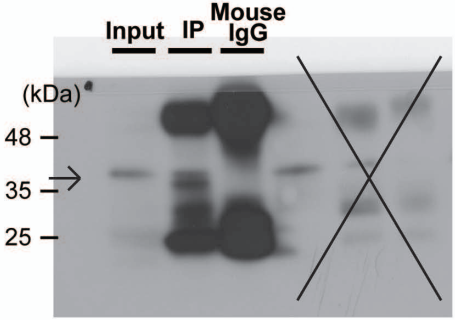

Fig 5B upper

IP: Sox2 Ab
WB: Sox2 Ab
(long exposure of Fig 5A)

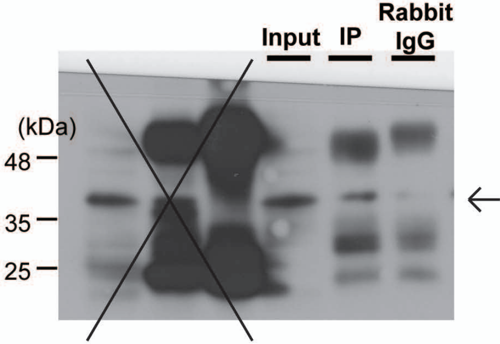

Fig 5B lower

IP: Sox2 Ab
WB: O-GlcNAc Ab
(RL-2)

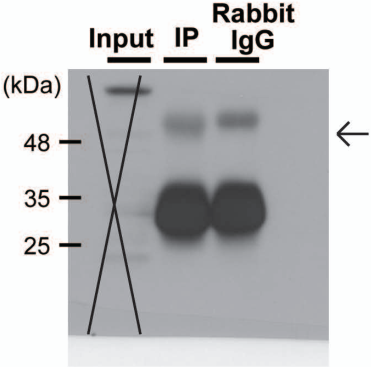

Fig 5C upper

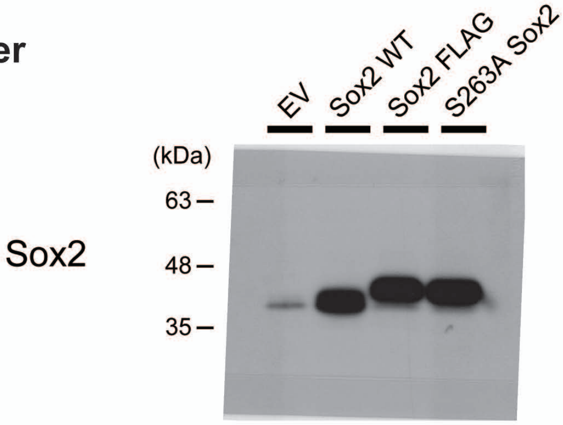

Fig 5C middle

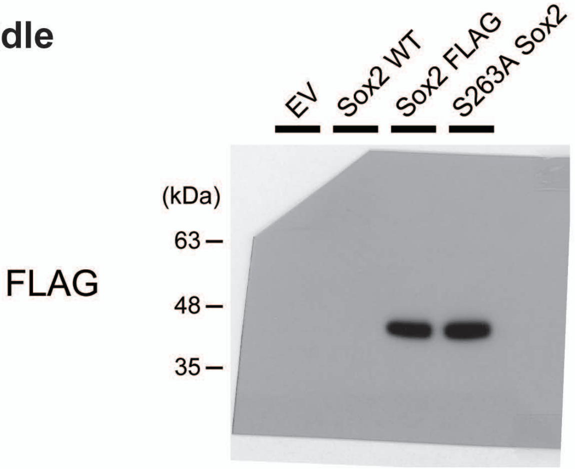

Fig 5C lower

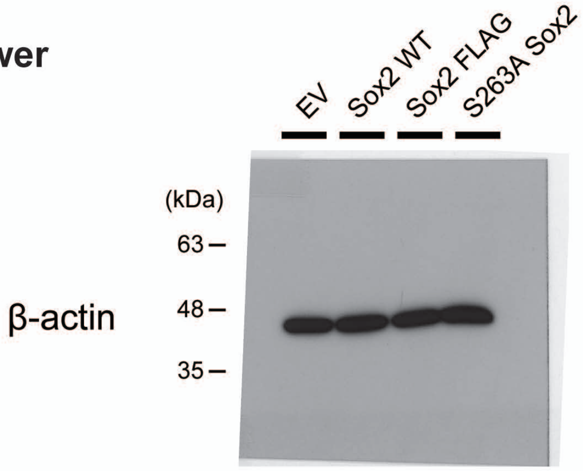

48 –

35 –

Fig 5D upper

IP: FLAG Ab
WB: Sox2 Ab
(Short exposure)

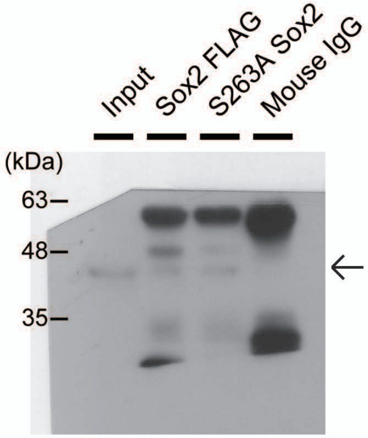

Fig 5D middle

IP: FLAG Ab
WB: Sox2 Ab
(Long exposure)

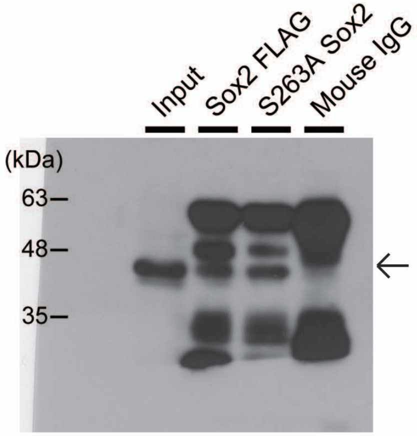

Fig 5D lower

IP: FLAG Ab
WB: O-GlcNAc Ab
(RL-2)

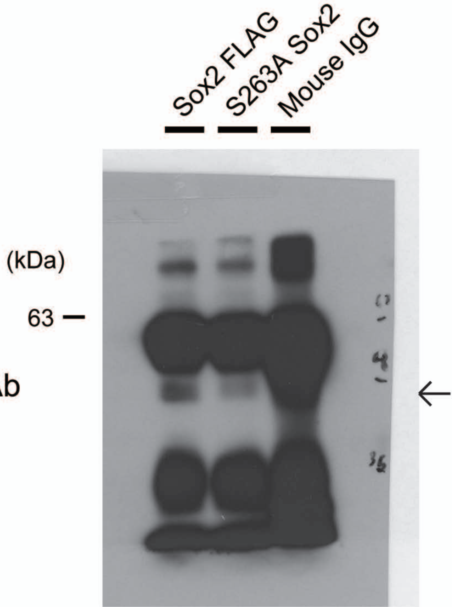

Supplement: S1 Raw images — (PDF) [file pone.0267804.s002.pdf]
